# Organomineral Fertilizer as Source of P and K for Sugarcane

**DOI:** 10.1038/s41598-020-62315-1

**Published:** 2020-03-25

**Authors:** Carlos Alexandre Costa Crusciol, Murilo de Campos, Jorge Martinelli Martello, Cleiton José Alves, Carlos Antonio Costa Nascimento, Júlio Cesar dos Reis Pereira, Heitor Cantarella

**Affiliations:** 1 São Paulo State University (UNESP), College of Agricultural Sciences, Dep. of Crop Science, Lageado Experimental Farm, P.O. Box: 237, Zip Code: 18610-034 Botucatu, São Paulo Brazil; 2 Sugarcane Technology Center (CTC), P.O. Box: 162, Zip Code:13400-970 Piracipaba, São Paulo Brazil; 3 Agronomic Institute of Campinas (IAC), Soils and Environmental Resources Center, Av. Barão de Itapura 1481, Zip Code: 13020-902 Campinas, São Paulo Brazil

**Keywords:** Biogeochemistry, Environmental sciences

## Abstract

Sugarcane (*Saccharum* spp) crop has high social, economic and environmental importance for several regions throughout the world. However, the increasing demand for efficiency and optimization of agricultural resources generates uncertainties regarding high mineral fertilizer consumption. Thereby, organomineral fertilizers are to reduce the conventional sources consumption. Thus, this study was carried out to evaluate the agronomic and economic sugarcane performancies and the residual effect of P and K under mineral and organomineral fertilization. Growth and technological parameters, leaf and soil nutrients concentration in surface and subsurface layers were analyzed from sugarcane planting (plant cane) until the first ratoon. Agronomic and economic sugarcane efficiency were evaluated. At the first ratoon, resin-extractable P provided by mineral and organomineral fertilizers were, respectively, 15 and 11 mg kg^−1^ in the 0.0–0.2 m, and 28 and 31 mg kg^−1^ in 0.2–0.4 m layer. However, exchangeable K in the 0.0–0.2 m layer was 1.88 and 1.58 mmol_c_ kg^−1^ for mineral and organomineral fertilizers, respectively. The yield gains over the control reached with mineral and organomineral fertilizers were, respectively, 10.99 and 17 Mg ha^−1^ at the lowest fertilizer rate; and 29.25 and 61.3 Mg ha^−1^ at the highest fertilizer rate. Agronomic and economic organomineral fertilizer efficiencies are more pronounced in plant cane. Summing two harvests, the organomineral is 7% more profitable than mineral fertilizer.

## Introduction

The sugarcane (*Saccharum* spp) crop is recognized worldwide for its high biomass production capacity, sequestering thousands of tons of atmospheric CO_2_ during its development, and it has a sustainable and very attractive balance related to greenhouse gas (GHG) emissions during its entire industrial process^1^. Thus, it is the main economically exploited crop in Brazil used for the production of clean and renewable energy, biofuel and sugar among other products^2^. In addition, the crop is responsible for creating millions of jobs and positively contributing to the environment^3,4^.

To meet the high nutritional demand of the crop, a great number of mineral fertilizers is commonly used to achieve satisfactory yields and sustainable revenue during all crop cycles. However, the increasing demand for efficiency and optimization of resources used during the agricultural production process generates uncertainties regarding the high mineral fertilizer consumption produced with imported raw material, substantially increasing the agricultural budget^5^.

In this sense, the recycling of agroindustrial organic waste appears as an alternative for substitution or supplementation of mineral fertilization for the sugarcane crop. Its reutilization in plant nutrition aiming for agricultural production is an excellent and sustainable form to supply the soil × plant × environment system demand^6^.

According to Raij^7^, the oldest fertilizers used in agriculture originated from organic sources, i.e. manure, sometimes referred to as natural fertilizers because of their origin. The agricultural use of organic waste constitutes an economically and environmentally viable practice mainly because it allows for the recovery of several chemical elements, such as nitrogen (N), phosphorus (P), potassium (K) and trace elements. In addition, it contributes through the addition of organic matter (OM) to the soil, improving the physical structure, water uptake capacity and nutrient supply to plants, thus increasing crop production^8^.

Low economic feasibility mainly because of the logistics of applying great amounts of organic fertilizers over large areas is an obstacle for organic compound use. The low analytical value of the different organic sources such as cow manure, vinasse and filter cake^9^ has forced the industry to enrich it with mineral soluble sources, providing high N, P, and K concentrations in a lower volume and creating so-called organomineral fertilizers. Thus, organomineral fertilizers are characterized as a mixture of organic and mineral fractions and can be produced in several N, P and K proportions suitable for crop requirements^1^. Usually, as a derivate to regional organic sources, the final product can be granulated, pelleted or powdered.

Recently, research efforts have been completed to evaluate the agronomic efficiency of fertilizers containing any organic compound. Mariano *et al*.^10^ evaluated the organomineral N application in sugarcane and observed comparable or superior biomass and nutrient content relative to mineral N fertilizers. Relating to P efficiency, different authors have used organic compounds associated with mineral P fertilizer and verified improvements in soil P availability, mainly because of the reduction in specific adsorption^11–13^. Regarding K, Rosolem *et al*.^14^ evaluated the efficiency of K sources using regular KCl and KCl coated with humic acid and concluded that coating KCl can control the release of K to the soil solution in light-textured soils, preventing losses via leaching.

The adoption of new agricultural practices, such as the use of organomineral fertilizers, depends on their efficiency and logistics, which can support the producers in obtaining higher yields and offer a sustainable and economically alternative in crop production^5^. Considering the sugarcane crop, however, most studies have only evaluated plant cane performance^15–17^. As sugarcane is considered a semi-perennial crop, there is a clear necessity to study the residual effect of the organomineral fertilization in ratoons as well, establishing scientific parameters that can drive and justify its use.

Therefore, the aim of this study was to evaluate the agronomic and economic sugarcane performance from the plant cane to first ratoon and the influence of the residual effect of P and K in surface and subsurface profiles under mineral and organomineral fertilization.

## Material and Methods

The experiment was performed in a commercial area of the Agrodoce Agricultural Group at Boracéia-SP, Brazil, during 2016 to 2018. The location of the experimental area is 22°11’ S and 48°48’ W at 480 m altitude. According to the Köppen classification, the predominant climate in the region is Cwa, which is mainly tropical humid with a hot summer. The soil was classified as a sandy-textured Typic Hapludox^18^. Chemical and physical characterizations^19^ were obtained from air-dried soil samples (0–0.20 and 0.20–0.40-m layers) passed through a 2-mm sieve (10 mesh) and showed the following results: Surface layer (sand, 705; clay, 48 and silt, 246 g kg^−1^); 5.6 pH (CaCl_2_); 16 g dm^−3^ organic matter; 9 mg dm^−3^ P_resin_; 1.1, 27, 10, and 15 mmol_c_ dm^−3^ of exchangeable K, Ca, Mg, and H + Al, respectively; and a base saturation (BS) of 71%. For the subsurface layer (sand, 702; clay, 41 and silt, 256 g kg^−1^); 5.3 pH (CaCl_2_); 10 g dm^−3^ organic matter; 14 mg dm^−3^ P_resin_; 0.54, 21, 07, and 18 mmol_c_ dm^−3^ of exchangeable K, Ca, Mg, and H + Al, respectively; and a base saturation (BS) of 61%.

A randomized block experimental design was established with four replicates using a 2 × 5 factorial scheme. The treatments consisted of two sources (mineral and organomineral fertilizers) applied at 5 rates as follows: 05–25–25 (N-P-K), commercial mineral fertilizer grade composed by monoammonium phosphate (MAP, 460 kg t^−1^), single superphosphate (SSP, 110 kg t^−1^) and muriate of potash (KCl, 430 kg t^−1^) at rates 0; 240 kg ha^−1^ (N, 12; P_2_O_5_, 60 and K_2_O, 60 kg ha^−1^); 480 kg ha^−1^ (N, 24; P_2_O_5_, 120 and K_2_O, 120 kg ha^−1^); 600 kg ha^−1^ (N, 30; P_2_O_5_, 150 and K_2_O, 150 kg ha^−1^) and 720 kg ha^−1^ (N, 36; P_2_O_5_, 180 and K_2_O, 180 kg ha^−1^) and Organomineral fertilizer, composed by MAP (288 kg t^−1^), KCl (250 kg t^−1^) and an granulated organic matrix (462 kg t^−1^)at rates of 0; 400 kg ha^−1^ (N, 12; P_2_O_5_, 60 and K_2_O, 60 kg ha^−1^); 800 kg ha^−1^ (N, 24; P_2_O_5_, 120 and K_2_O, 120 kg ha^−1^); 1000 kg ha^−1^ (N, 30; P_2_O_5_, 150 and K_2_O, 150 kg ha^−1^) and 1200 kg ha^−1^ (N, 36; P_2_O_5_, 180 and K_2_O, 180 kg ha^−1^). These treatments were established in February 2016 (plant cane) and the fertilizers were applied in the planting furrow bottom, 0.2–0.3 m deep. For 2018 (first ratoon), a single rate of 170 kg ha^−1^ for N and K was applied for all plots aiming to supply the nutrient exportation by the mean sugarcane yield of the first crop season and not to be an interference factor in the ratoon yield, making it possible to evaluate the residual effect of the applied fertilizer on the cane plant. As source of N and K were used KCl (333 kg t^−1^) and ammonium nitrate (606 kg t^−1^) and an organomineral [composed by urea (325 kg t^−1^), KCl (233 kg t^−1^) and a granulated organic matrix (442 kg t^−1^)] for mineral and organomineral plots, respectively. Each plot consisted of 4 double-rows (2.4 × 0.9 m) 20 m in length disregarding the 0.5-m edge at each end.

The organomineral fertilizer is a commercial grade fertilizer manufactured at Solvi group fertilizer industry located in Coroados, São Paulo, Brazil (21°23'07.1”S 50°15'15.0”W), commercialized under the name of Organosolvi^®^ and is openly available to customers on www.organosolvi.com. The organic matrix of the organomineral is originated from the agroindustries located near the fertilizer plant, and is composed by byproducts of meat industry (rumen, blood, bones), dairy products, Fuller’s earth (high absorbent and high CEC clay minerals used in the tallow industry), pine bark and eucalyptus. Before its use for organomineral production, it is submitted to a composting process. After composting its chemical composition showed the following results, in g kg^−1^, 177 of OC, 23 of N, 16 of P_2_O_5_, 18 of K_2_O, 23 of Ca, 5.0 of Mg, 5.0 of S and, in mg kg^−1^, 148 of Zn, 27 of Cu, 416 of Mn, 10.000 of Fe, 600 of B and a moisture of 24.2%, pH (CaCl_2_) = 8.1 and CEC = 607.2 mmol_c_ kg^−1^. Then, the compost is sent to a rotating drum that consists of shaping and sphericity, and then to a rotary dryer with a hot air flow (120 °C). After drying, the granules were classified in sieves of 1 to 4 mm, acquiring a granulometry very similar to the standard mineral fertilizer. Following this process, it was mixed with mineral fertilizers (MAP, KCl and urea treated with a nitrification inhibitor + polymer-based additive that controls the N availability and minimizes N losses via volatilization). The nitrogen sources used in mineral fertilization (MAP for plant cane and ammonium nitrate for ratoon) are not likely to lose N by volatilization due to the low pH resulted of their dissolution^20–23^. Thus the N amount supplied by both organomineral and mineral fertilizers would be the same, allowing to evaluate the organic matter matrix effect regardless the N losses from nitrogen sources. Urea has a high N content (45%), thus this is the best N source for organomineral fertilizer because it allows increasing the N content in it with the smallest increment in its final mass.

Following 6 months of treatment application, a period of full vegetative growth of sugarcane, 10 ^+^1 leaves or TVD (Top Visible Dewlap leaf), were collected within each plot in the two central rows according to the numbering system suggested by Kuijper^24^. Disregarding the leaf midrib and considering only the middle third of the leaf blade, the material was dried in an oven with forced air circulation at 60 °C until a constant mass was obtained. It was then milled and leaf N, P and K contents were determined^25^. Prior to harvest, the stalk number m^−1^ was determined by counting the stalks in the two central rows within the useful area of each plot and then converting to the number of stalks m^−1^. Stalk weight, diameter, plant height, internode number and length were calculated as the means of the ten stems collected from each plot, clipped at the apical bud height, defoliated and measured using a digital scale, caliper and a ruler marked in meters from the soil surface up to the auricle region of the +1 or TDV leaf. After the growth evaluations, the cleaned stalks were sent to the Middle Tietê Sugarcane Planters Association (ASCANA) Laboratory, in Lençóis Paulista, SP, Brazil for processing according to the methodology defined in the Sucrose Content-Based Sugarcane Payment System, in accordance with Consecana’s semiannual updates for the technological evaluations as described by Fernandes^26^. At harvesting, the four central rows of each plot were mechanically harvested and stalks were weighed using an electronic load cell. Then, the stalk yield was estimated extrapolating the values to stalk yield ha^−1^, disregarding planting holes (gaps greater than 0.5 m). Sugar yield ha^−1^ was estimated as the product of the multiplication of the sucrose concentration (%) and stalk yield (Mg ha^−1^) at harvest. Following harvest, soil samples were collected at depths of 0.00–0.20 and 0.20–0.40 m in all plots using a Dutch auger. Five subsamples were randomly collected in planting row of each plot and combined into a composite sample. Soil chemical attributes were determined following the method of Raij *et al*.^19^.

The agronomic efficiency index (AEI)^27^ and the economic efficiency index (EEI) were calculated as the percentage ratio between the stalk and sugar yields (AEI) and net profits (EEI) resulting from the mineral and organomineral fertilizers applied at the same rate in the plant cane and the residual effect in first ratoon. For AEI, the crop yield obtained in the treatment control was subtracted from both yields as follows (Eq. 1):

1 $$\mathrm{AEI}( \% )=[({\rm{Y}}2-{\rm{Y}}1)/({\rm{Y}}3-{\rm{Y}}1)]\times 100$$

where Y1 = crop yield in the control treatment; Y2 = crop yield using organomineral fertilizer at the corresponding rate; and Y3 = crop yield with mineral fertilizer at the corresponding rate.

For EEI, the percentage ratio of the net profits obtained for both fertilizers was obtained by Eq. 2:

2 $${\rm{EEI}}( \% )=({\rm{X}}2/{\rm{X}}1)\times 100$$

where X2 = net profit using organomineral fertilizer at the corresponding rate and X1 = net profit using mineral fertilizer at the corresponding rate. The net profit was calculated using the Eq. 3:

3 $${\rm{Net}}\,{\rm{profit}}(\text{US}\$\,{{\rm{ha}}}^{-1})={\rm{Revenue}}\,\mbox{--}\,{\rm{Fertilizers}}\,{\rm{cost}}$$

The revenue and fertilizers cost were converted using dollar quotation at each respective time (harvests, September 2017 and 2018; planting fertilization, February 2016 and first ratoon fertilization, October 2017). The revenue was obtained using the Eq. 4:

4 $${\rm{Revenue}}(\text{US}\$\,{{\rm{ha}}}^{-1})=(({\rm{Value}}\,{\rm{of}}\,{\rm{TRS}}\times {\rm{sugarcane}}\,{\rm{TRS}})\times ({\rm{stalk}}\,{\rm{yields}}))$$

The total economic efficiency index (EEI total) was estimated similarly to the EEI, but using the sum of net profit of plant cane and first ratoon.

Data for each crop season were separately subjected to analysis of variance. The fertilizer source means were compared using the t test (LSD) at 5% probability. The rate effects were evaluated via regression analysis using the statistical software package SISVAR^28^.

## Results

### Plant cane

**Figure 1:**
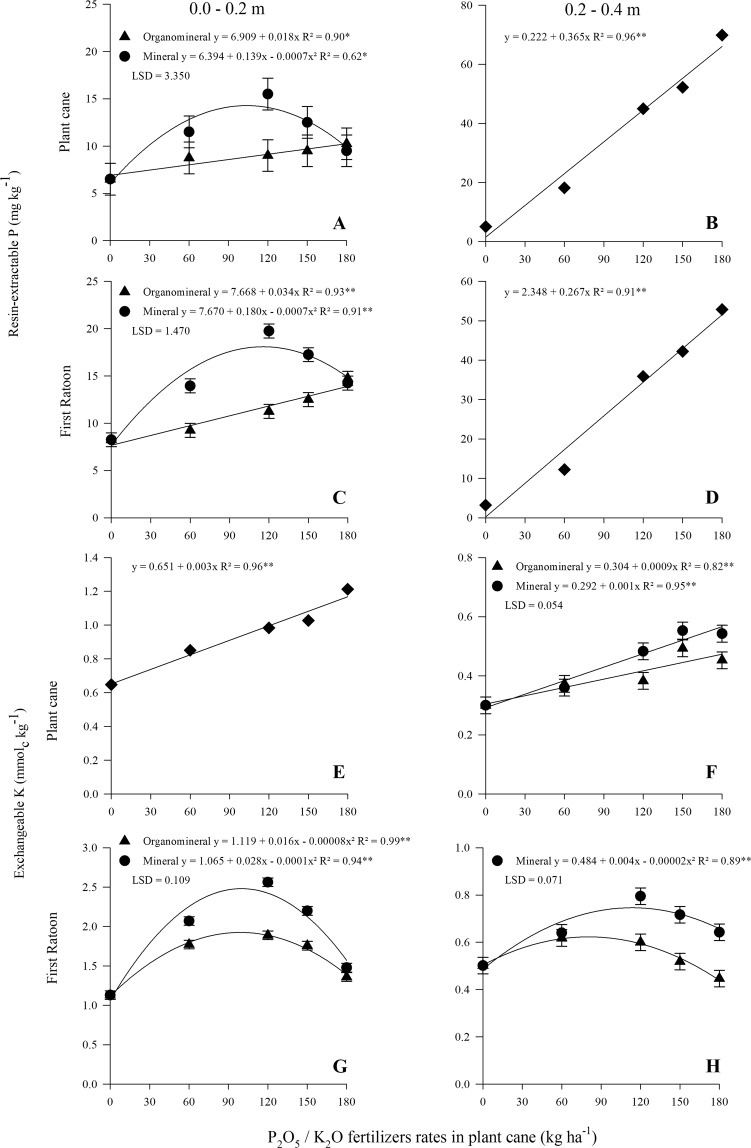
Soil resin-extractable P in plant cane (A. 0.0–0.2 m and B. 0.2–0.4 m) and first ratoon (C. 0.0–0.2 m and D. 0.2–0.4 m) and effect of soil exchangeable K in plant cane (E. 0.0–0.2 m and F. 0.2–0.4 m) and first ratoon (G. 0.0–0.2 m and H. 0.2–0.4 m) as function of mineral and organomineral P_2_O_5_/K_2_O fertilizers rates application. Bars represent LSD (least significant difference) for fertilizers within same dose at 5% of probability. Single fit means no interaction between fertilizer and rates.

There was significant interaction between the sources and rates for the resin-extractable P content in the surface layer (Table 1; Fig. 1A). The mathematical adjustment for the mineral fertilizer was quadratic, while in the organomineral fertilizer it was linear, with a significant difference only when applying 120 kg ha^−1^ of P_2_O_5._ Related to the subsurface layer, soluble P content significantly varied only as a function of the rates of P_2_O_5_ (Table 1; Fig. 1B).

**Table 1:** Soil resin-extractable P and exchangeable K content as a function of mineral and organomineral fertilizer rates after harvests related to plant cane and first ratoon, Boracéia, SP, 2017 and 2018.

Treatments	P mg kg^−1^		K mmol_c_ kg^−1^	
	Plant cane	First ratoon	Plant cane	First ratoon
	**0.0–0.2 m**			
Mineral	9.2	15a	0.95	1.88a
Organomineral	8.5	11b	0.94	1.58b
F Probability				
Source (S)	0.0578	<0.0001	0.4969	<0.0001
Rates (R)	0.0008	<0.0001	<0.0001	<0.0001
SxR	0.0131	<0.0001	0.5611	<0.0001
SE^(a)^	1.1570	0.5069	0.0128	0.0392
CV (%)^(b)^	24.29	7.83	6.08	4.52
	**0.2–0.4 m**			
Mineral	38a	28b	0.45a	0.65a
Organomineral	36a	31a	0.40b	0.53b
F Probability				
Source (S)	0.0717	0.0022	0.0007	<0.0001
Rates (R)	<0.0001	<0.0001	<0.0001	<0.0001
SxR	0.5338	0.1474	0.0213	0.0003
SE^(a)^	1.4759	1.2400	0.0087	0.0257
CV (%)^(b)^	7.96	8.37	9.25	8.62

^(a)^Standard Error. ^(b)^Variation Coefficient.

Means followed by equal letters, in the lines, do not differ significantly by the LSD test, at 5% probability.

The exchangeable K content in the surface layer linearly increased as a function of P and K fertilizer rates, without differences between sources (Table 1; Fig. 1E). For the subsurface layer, a linear interaction effect was observed between the sources and rates, being different at 120 and 180 kg ha^−1^ of K_2_O (Table 1; Fig. 1F).

There was not a significant change in the N, P and K leaf concentration (Table 2). On average, the values for N (18–25 g kg^−1^) and P (1.5–3.0 g kg^−1^) were within the reference limit for sugarcane^29,30^. K (10–16 g kg^−1^), although showing a similar value, is below the lower boundary range^29,30^.

**Table 2:** N, P and K leaf concentrations and biometric data as a function of mineral and organomineral fertilizer rates in plant cane and first ratoon, Boracéia, SP, 2017 and 2018.

Treatments	N		P		K		Plant Height (m)		Internodes plant^−1^	
	Plant cane	First ratoon	Plant cane	First ratoon	Plant cane	First ratoon	Plant cane	First ratoon	Plant cane	First ratoon
**Source**	**g kg**^**−1**^									
Mineral	20	15a	1.7	1.6a	9.3	10.6b	2.5b	2.7	17b	20
Organomineral	20	13b	1.8	1.5b	9.0	11.4a	2.6a	2.7	19a	20
F Probability										
Source (S)	0.4910	<0.0001	0.2706	<0.0001	0.4944	0.0034	0.0154	0.3658	<0.0001	0.7917
Rates (R)	0.6597	<0.0001	0.0936	<0.0001	0.0540	<0.0001	0.0592	0.0626	0.0392	0.3609
SxR	0.9624	<0.0001	0.3949	0.0016	0.8324	0.3119	0.3560	0.4561	0.0204	0.8940
SE^(b)^	0.3367	0.1533	0.0304	0.0131	0.3067	0.1904	0.0289	0.0226	0.2045	0.1723
CV (%)^(c)^	7.48	4.82	7.69	3.73	14.92	7.61	5.67	3.77	5.12	3.78
	**Diameter (mm)**		**Stalk m**^**−1**^		**Stalk Yield Mg ha**^**−1**^		**Pol (%)**^**(a)**^		**Sugar Yield Mg ha**^**−1**^	
Mineral	28	31	7.9	8.9	113b	114b	14.1	16.3	16b	19
Organomineral	28	31	8.1	8.9	123a	117a	14.4	16.1	18a	19
F Probability										
Source (S)	0.7924	0.7973	0.1637	0.5689	<0.0001	0.0456	0.0768	0.1472	<0.0001	0.5097
Rates (R)	0.2098	0.0696	0.3561	0.2140	<0.0001	0.0044	0.9356	0.8270	<0.0001	<0.0001
SxR	0.3374	0.6725	0.2541	0.5304	<0.0001	0.8204	0.4217	0.4672	<0.0001	0.9001
SE^(b)^	0.2659	0.2071	0.0612	0.1011	1.7957	1.0967	0.0955	0.0658	0.2850	0.1919
CV (%)^(c)^	4.28	2.65	3.47	5.08	6.83	4.22	2.99	1.83	7.58	4.58

^(a)^Apparent Sucrose: the amount of sucrose in a sugar product. ^(b)^Standard Error. ^(c)^Variation Coefficient.

Means followed by equal letters, in the lines, do not differ significantly by the LSD test, at 5% probability.

**Figure 2:**
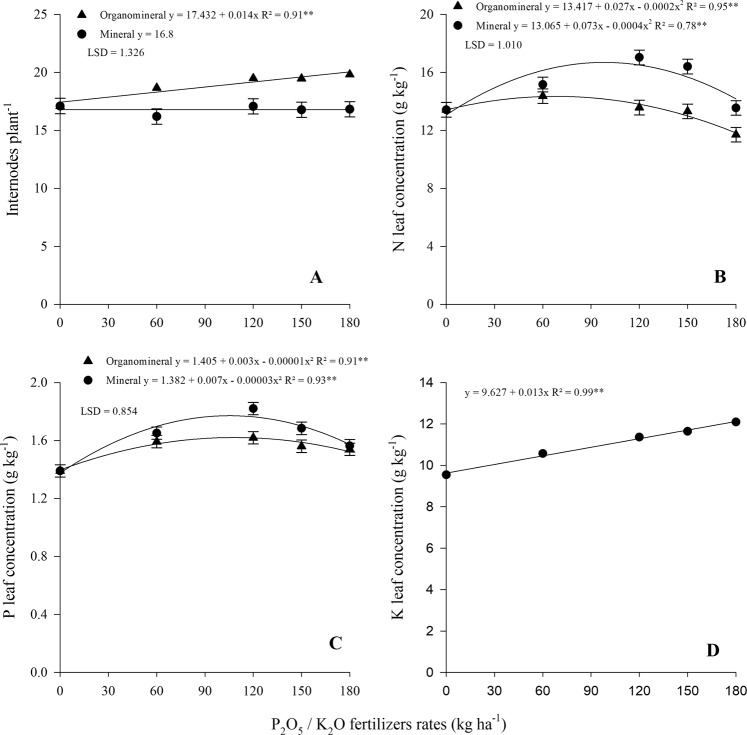
Internodes per plant in plant cane (A), N (B), P (C) and K (D) leaf concentration in the first ratoon as function of mineral and organomineral P_2_O_5_/K_2_O fertilizers rates application. Bars represent LSD (least significant difference) for fertilizers within same dose at 5% of probability. Single fit means no interaction between fertilizer and rates.

Related to growth parameters, on average, plant height was significantly higher where organomineral fertilizer was applied (Table 2). For internodes per plant, the interaction effect between sources and rates was significant only for the organomineral fertilizer, linearly increasing as a function of fertilizer rates (Fig. 2A).

**Figure 3:**
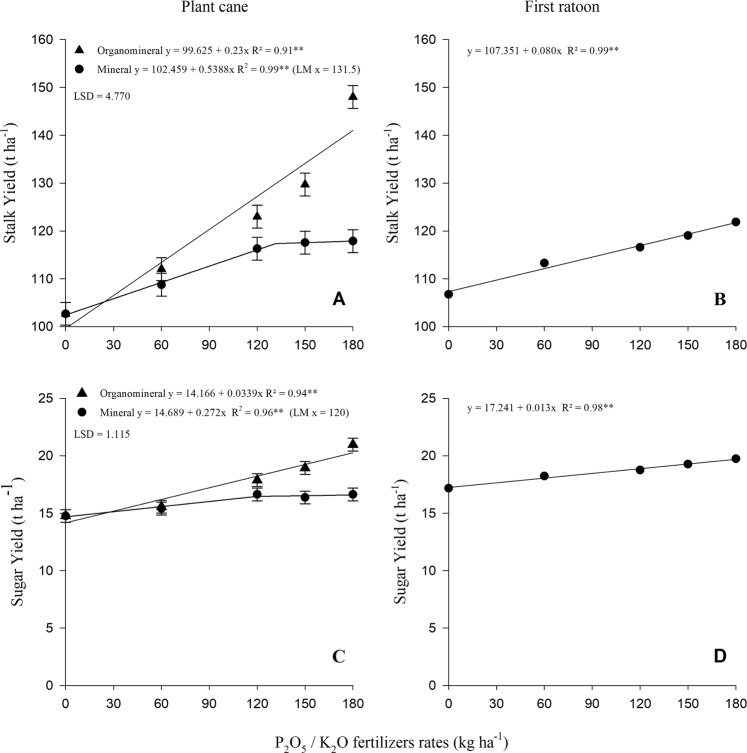
Stalk (A in plant cane and B in the first ratoon) and sugar yield (C in plant cane and D in the first ratoon) as function of mineral and organomineral P_2_O_5_/K_2_O fertilizers rates application. LM - line meeting (the value of x where the lines intersect in graphics B and C). Bars represent LSD (least significant difference) for fertilizers within same dose at 5% of probability. Single fit means no interaction between fertilizer and rates.

There was a significant interaction effect between sources and rates for stalks (Fig. 3A) and sugar yield (Fig. 3C). For both parameters, the adjustments were linear, showing a response until the highest applied rate for organomineral fertilizer. For the mineral fertilizer, the adjustments were linear until the estimated rate at 132 and 120 kg ha^−1^ P_2_O_5_/K_2_O for stalks and sugar yield, respectively, stabilizing for the two last rates. On average, the organomineral fertilizer produced 9% more yield than the mineral fertilizer.

The AEI followed the same trend of stalk and sugar yield results (Table 3). Organomineral fertilizer provided greater increases in stalk and sugar yields than those of the mineral fertilizer for all P and K rates, especially at the highest rate, being, on average, 98 and 113% more efficient, respectively. In spite of the higher fertilization cost, organomineral fertilizer was, on average, 12% more profitable when compared to the standard mineral fertilizer. The higher P_2_O_5_/K_2_O rate (180 kg ha^−1^) was 27% more profitable.

**Table 3:** Increased yield of stalks (IStY) and sugar (ISgY), agronomic efficiency index of stalks (AEIst) and sugar (AEIsg), economic efficiency index (EEI) and total economic efficiency index (EEI total) of of stalks yield as a function of P_2_O_5_/K_2_O sources and rates.

P_2_O_5_/K_2_O (kg ha^−1^)	IStY (Mg ha^−1^)^(a)^		AEIst (%)^(b)^	ISgY (Mg ha^−1^)^(c)^		AEIsg (%)^(d)^	Revenue (US$ ha^−1^)^(e)^		Cost (US$ ha^−1^)^(f)^		Net profit (US$ ha^−1^)^(g)^		EEI (%)^(h)^	EEI total (%)^(i)^
	Min	Org	Org	Min	Org	Org	Min	Org	Min	Org	Min	Org	Org	Org
2017														
0	—	—	—	—	—	—	2674	2674	—	—	2674	2674	—	—
60	6.07	9.34	154	0.66	0.81	124	2813	2938	87	133	2726	2805	103	102
120	13.60	20.28	149	1.88	3.12	166	3007	3247	174	266	2833	2981	105	103
150	14.86	26.99	182	1.62	4.18	258	2976	3448	218	333	2758	3115	113	108
180	15.19	45.30	298	2.04	6.21	304	3005	3881	261	400	2744	3481	127	114
Mean	—	—	196	—	—	213	—	—	—	—	—	—	112	107
2018														
0	—	—	—	—	—	—	2389	2389	70	82	2319	2307	—	—
60	4.92	8.36	170	1.09	1.05	97	2531	2545	70	82	2461	2463	100	—
120	8.16	11.68	143	1.49	1.70	114	2588	2634	70	82	2518	2552	101	—
150	9.84	14.75	150	1.79	2.43	136	2626	2720	70	82	2556	2638	103	—
180	14.06	16.00	114	2.57	2.58	100	2738	2748	70	82	2668	2666	100	—
Mean	—	—	144	—	—	112	—	—	—	—	—	—	101	—

^(a)^Increase in stalks yield relative to the mean yield in control; ^(b)^Agronomic efficiency index of the organomineral relative to the mineral fertilizer in stalks yield; ^(c)^Increase in sugar yield relative to the mean yield in control; ^(d)^Agronomic efficiency index of the organomineral relative to the mineral fertilizer in sugar yield; ^(e)^Revenue (US$ ha^−1^) = ((Value of TRS (US$ kg^−1^) × sugarcane TRS (kg Mg^−1^)) × stalk yield (Mg ha^−1^)); ^(f)^Costs of fertilizers (US$ ha^−1^) = Fertilizers price × respective applied rate; ^(g)^Net profit (US$ ha^−1^) = Revenue – Costs; ^(h)^Economic efficiency index (EEI) of the organomineral in relation to the mineral fertilizer in profitability in the stalk yield; ^(i)^Total Economic efficiency index (EEI total) is the sum of EEI in 2017 + 2018.

*Average dollar value (february/2016 − US$ 1.00 is equivalent to R$ 3.99; september/2017 – US$ 1.00 is equivalent to R$ 3.16; november/2017 − US$ 1.00 is equivalent to R$ 3.40 and September 2018 – US$ 1.00 is equivalent to R$ 4.03. Available in www.bcb.gov.br.

**Total Reducing Sugars (TRS) (September/2017 – US$ 0.1821 kg^−1^ of TRS and September 2018 – UU$ 0.1399 kg^−1^ of TRS. Available in www.consecana.com.br.

### First ratoon

Related to the soil residual effect of P, there was significant interaction between the sources and rates for soluble P content in the surface layer (Table 1). Following the same trend as that of the plant cane, the adjustment in the soil soluble P content was quadratic for the mineral fertilizer application and linear for the organomineral fertilizer (Fig. 1C). The mineral fertilizer was higher up to 150 kg ha^−1^ P_2_O_5_ with no significant difference at the highest rate (Fig. 1C). Related to the subsurface layer, the soil soluble P content positively ranged as a function of P_2_O_5_ rates (Fig. 1D). For P_2_O_5_ sources, on average, soil soluble P content was statistically higher using organomineral fertilizer. Because P_2_O_5_ was not applied in the first ratoon, the P content was smaller than the plant cane evaluation, as expected because of the sugarcane uptake.

For the exchangeable K content, in both layers, a significant interaction between sources and rates were observed with a quadratic adjustment (Table 1). In both cases, the exchangeable K content was higher when the mineral K fertilizer was applied and, in the surface layer, the interaction was not significant only at the highest K_2_O rate (Fig. 1G). Regarding to subsurface layer, there was not a significant difference in exchangeable K content only up to 60 kg ha^−1^ of K_2_O above which it was higher with the mineral fertilizer application for the other three higher K_2_O rates (Fig. 1H).

There was significant polynomial interaction effect between the sources and rates for the N and P leaf concentration (Table 2). The mathematical adjustment was quadratic for both (Fig. 2B,C). Related to N, there was no significant difference for the first rate applied; however, for the other rates, the N uptake was higher using the mineral fertilizer. In spite of this, the N leaf concentration was less than the lower boundary range (18–25 g kg^−1^). The P leaf concentration was also higher using mineral fertilizer at rates equivalent to 120 and 150 kg ha^−1^ P_2_O_5._ Only the P leaf concentration in the treatment control was below the range considered optimal for sugarcane (1.5–3.0 g kg^−1^). The K leaf concentration linearly increased as a function of the applied rates, without differences between the sources (Fig. 2D). Similar to P, only in the control was the K leaf concentration less than the range considered optimal for sugarcane (10–16 g kg^−1^).

A significant difference was not observed related to growth parameters; however, stalks and sugar yield linearly increased as a function of fertilizer rates (Table 2; Fig. 3B,D). For stalk yield, as well, there was a significant difference related to the sources, being, on average, 3% higher with organomineral fertilizer application (Table 2). The AEI (Table 3) for both parameters was lower than the plant cane. On average, the increase in stalk and sugar yields was 44 and 12% respectively higher than mineral source. Nevertheless, the EEI was practically the same, not showing economic gains at the ratoon. Considering the sum of the two evaluated harvests, the use of organomineral fertilizer proved to be more profitable (7%, on average), mainly in the higher rate of P and K (14% at rate of 180 kg ha^−1^ of P_2_O_5_/K_2_O) (Table 3).

## Discussion

Soil soluble P in the surface layer, was higher in the treatments with mineral fertilizer application with quadratic distribution of the points (Fig. 1A,C). However, soil P content with organomineral fertilization linearly increases, without a difference in the mineral P at the highest P_2_O_5_ rate. This difference in P content may be related to its solubility. The P from the mineral fertilizer is readily soluble, detectable in the resin-extractable P analysis; otherwise, organomineral P depends on soil mineralization for availability, demanding an OM-P linkage breaking from the organic structure, which can be characterized as slow P release^31,32^.

Because the soil P application occurred in the planting furrow, the highest soil P content was in the subsurface layer, linearly increasing as a function of the P_2_O_5_ rates. Although there was no P application in the first ratoon, the P content remained high because of its soil residual effect. It is likely the increase in the stalks and sugar yields was related to the increase in soil P content in the subsurface layer, because P plays an important role in sugarcane rooting and tillering, positively affecting stalk and sugar yields^33,34^. Similarly, in this study, other authors have shown that mineral fertilizer, when associated with any organic compound, can be more efficient mainly by the reduction in P adsorption onto Al and Fe minerals^11,35,36^. Despite soil resin-extractable P content in organomineral fertilizer treatments have been higher, it should be emphasized that this study was conducted in a largely sandy-textured soil, in which P adsorption is naturally reduced^36,37^ allowing high resin-extractable P content regardless of the P_2_O_5_ source. Thus, stalks and sugar yield in first ratoon were not affected by these variables. Besides the effects of the organomineral organic matrix on reducing P adsorption; the addition of organic matter to the soil may help to maintain its moisture near fertilizer placement for more time which favor the soil P diffusion^38,39^, and increases the P amount reaching the roots.

In the plant cane, although there were linear increases in soil K content as a function of the K_2_O rates for both sources, there was no difference in the K leaf concentration. In relation to the first ratoon, the maximum estimated soil K content for the first layer was obtained at the rate of 100 kg ha^−1^ of K_2_O (mineral fertilizer) and 99 kg ha^−1^ of K_2_O (organomineral fertilizer). Although this dose is practically the same, a higher solubility for the mineral fertilizer was noted, because the quantified soil level was, respectively, 2.4 and 1.9 mmol_c_ kg^−1^. The high CEC value, quantified to the OM portion of the organomineral fertilizer, may adsorb part of the K applied, controlling its solubility in the soil. Rosolem *et al*.^14^ observed a higher efficiency with humic substances coating KCl when the fertilizer was applied in a single dose. They attributed this to the slow release because of the high CEC of the humic and fulvic acids. For perennial crops, such as sugarcane, this process can aid in the gradual nutrient release and decrease potential losses via leaching and runoff^37^. The higher P concentration in soil subsurface is due to the fertilizers were applied in the planting furrow (0.20–0.30 m depth). The higher K content on the surface is due to the fact that sugarcane straw releases more than 50 kg K_2_O ha^−1^ ^40^. Even in the sugarcane plant, where the amount of straw on the soil is much smaller, there may be a contribution of K present in the leaves that fall on the soil as the plant grows, since the release of K by the straw is relatively fast. Oliveira *et al*.^41^ observed that the sugarcane straw released 85% of its K content during the first year following sugarcane harvest.

Although the soil K content showed a quadratic adjustment, in the leaf, the increase was linear. The linear increase, also observed in stalk and sugar yields, may indicate there was higher K uptake by the plant, justifying the decrease in soil K content at the highest K_2_O rates. As well reported by Almeida *et al*.^42^, the increase in sugar yield by K_2_O rates may be related to the increase in stalk yield and K leaf concentration, because K acts in the transport via the phloem and carbohydrate storage^43^.

There was no variation in the N, P and K leaf concentration in the plant cane. Notably, there was source efficiency allowing the culture to maintain its nutritional status within the proper range. In addition, as the sugarcane was planted in a conventional soil tillage system, with rotation of the soil arable layer, there is natural organic matter mineralization, releasing nutrients to the plants and contributed to their nutrition^44^.

In the first ratoon, the range of N, P and K leaf concentrations among the treatments did not alter the stalks and sugar yield. For N, even considering that the organomineral source uses a urease enzyme polymer inhibitor, the N leaf concentration was less than that of the treatments using mineral fertilization and both were below the range considered optimal for sugarcane. The quadrac fit find for N and P concentration in leaf may be due to antagonism between K and N as well as between Cl and P. Considering that the source of K in both fertilizers was mainly muriate of potash (KCl). However, while the effect of K on N absorption is well known and accepted;^45,46^ the antagonism between Cl and P remains uncertain^47,48^. Also, quadratic behavior of both nutrients may be caused by the dilution effect, that is biomass accumulation is constant while nutrient absorption rate is reduced^49,50^.

The AEI (Table 3) showed a sugarcane positive response in both harvests, but more pronounced in plant cane. Similarly, De Souza^15^ concluded that in 90% of evaluated areas, organomineral fertilizer promoted better sugarcane performance, mainly in plant cane. Teixeira *et al*.^16^ also reported higher efficiency in stalk and sugar yield in plant cane using organomineral fertilizer at the highest rate, obtaining the same stalk yield with approximately 30 kg ha^−1^ less P_2_O_5_ fertilizer. Ramos *et al*.^17^ also observed a higher stalk yield applying organomineral fertilizer compared to poultry litter and mineral fertilizer in plant cane, recommending it for sugarcane. Because of intense soil tillage for sugarcane establishment, the soil organic fraction is stimulated to mineralize and organomineral fertilizer or other organic sources, e.g., filter cake, can contribute to higher agronomic stability^31^ as reflected in higher yields.

Regarding the first ratoon, perhaps sugarcane straw can influence mineral fertilizer efficiency. Satiro *et al*.^51^ evaluated sugarcane straw removal’s effects on soil degradation in the first and second ratoons and concluded that the impacts on soil chemical attributes were significant, mainly in sandy-textured soil. Because in this study sugarcane straw was not removed from the area, OM additions could contribute to a more balanced soil environment. However, further long-term field-scale research of differently textured soils is needed to explore in depth organomineral fertilizer performance for all planned sugarcane ratoons, providing more information regarding the soil residual effect of the nutrients and their influence on the stalk and sugar yield.

The higher efficiency of organomineral fertilizer, added to indirect parameters related to organic compounds reported in the literature, such as the slow release effect of nutrients, adequate soil biological activity, improvements in physical and chemical soil quality, better water retention capacity and soil porosity^32^, perhaps can explain the better sugarcane performance in this study.

Economically, in plant cane, in spite of the higher cost of production, due to the higher amount applied with organomineral, the increase in stalks and sugar yield guaranteed higher net profit (12%) compared to the mineral fertilizer. However, in the first ratoon, the profitability of both sources was equivalent. Considering the sum of the net profit in the two harvests, a greater profitability was obtained with the use of the organomineral fertilizer (7%), which justifies its use.

Considering these results, organomineral fertilizer is a promising agronomic and economic alternative for sugarcane producers. Cherubin *et al*.^31^ highlighted the importance of fertilizer management strategies including a balance between organic and mineral P sources to improve the yield, soil quality, and environmental sustainability of Brazilian sugarcane production. However, some concerns such as the concentration of potentially toxic elementssuch as plumbum (Pb) and arsenic (AS)^52^, availability of the required amount and logistics for large areas should be considered.

## Conclusions

Organomineral fertilizer is suitable to supply sugarcane requirements and can completely replace mineral fertilizer. However, its influence on sugar yield is lower than on stalks yield. In addition, organomineral fertilizer efficiency in stalks and sugar yield is more pronounced in plant cane, being, on average, 96 and 113% more efficient than mineral fertilizer, respectively. Consequently, organomineral fertilizer is more economically efficient in plant cane, being, on average, 12%. In the two harvests summed, the organomineral is 7% more profitable than mineral fertilizer.

For plant cane, mineral fertilizer provide higher P and K soil concentrations than organomineral fertilizer. This effect is mainly in the surface layer for P and in both layers for K. Also, there is higher residual P availability using organomineral fertilizer, however, stalks and sugar yield are not affected.
